# Comparison of high-intensive and low-intensive electromechanical-assisted gait training by Exowalk**®** in patients over 3-month post-stroke

**DOI:** 10.1186/s13102-022-00515-0

**Published:** 2022-07-10

**Authors:** Chang Seon Yu, Yeon-Gyo Nam, Bum Sun Kwon

**Affiliations:** 1 Department of Rehabilitation Medicine, School of Medicine, Dongguk University, Seoul, 04620 Republic of Korea; 2 Institute of Posture Science, School of Medicine, Dongguk University, Gyeongju, Republic of Korea; 3 Department of Rehabilitation Medicine, Dongguk University Ilsan Hospital, 27, Dongguk-ro, Ilsandong-gu, Goyang-si, Gyeonggi-do, 10326 Republic of Korea

**Keywords:** Gait, Exoskeleton device, Rehabilitation, Stroke

## Abstract

**Background:**

This study was conducted to assess the effect of electromechanical-assisted gait training intensity on walking ability in patients over 3-month post-stroke.

**Methods:**

Data from two randomized controlled trials (RCTs) were collected under the same study design of assessment and intervention, excluding intervention time per session. After matching the inclusion criteria of two RCTs, the experimental groups of each RCT were defined as low-intensive (LI) and high-intensive (HI) group according to the intervention time per session. Primary outcome was the difference of the change in Functional Ambulatory Categories (FAC) between LI and HI gait training. Secondary outcomes were the difference of changes in mobility, walking speed, walking capacity, leg-muscle strength, balance and daily activity evaluated with Rivermead Mobility Index (RMI), 10 m walk test (10MWT), 6-min walk test (6MWT), Motricity Index (MI), Berg Balance Scale (BBS) and Modified Barthel Index (MBI) respectively.

**Results:**

The FAC improved after gait training in both groups. The secondary outcomes also improved in both groups except RMI and MI in HI group. The change of all outcomes were not different between groups except RMI. The change of RMI in the LI group was greater than that in the HI group statistically, but it did not meet minimal clinically important difference.

**Conclusions:**

The improvement of walking ability after LI or HI gait training was not different if providing the same total gait training time. By providing the electromechanical gait training intensively, we could shorten the gait training period to improve walking ability and customize the training program according to the patient training abilities.

***Trial registration*:**

Name of the registry: Clinical Research Information Service. Trial registration number: No. KCT0002195(RCT1), No. KCT0002552(RCT2). Date of registration: 10/04/2016(RCT1), 10/05/2017(RCT2). URL of the trial registry record: https://cris.nih.go.kr/cris/search

## Background

Electromechanical-assisted gait training for stroke patients has been rapidly developed in recent years, and is being used as a new method of rehabilitation [1]. It has been reported in many studies as a treatment option to replace or supplement conventional rehabilitation by means of focused, repetitive, and active motions for stroke patients [2–6]. For the clinical effects of electromechanical-assisted gait training, Mehrholz et al. [6] demonstrated that it could improve post-stroke independent walking recovery when combined with physical therapy in patients suffering from a stroke. However, the most effective frequency, duration, and timing of post-stroke robot-assisted gait training are still unresolved [1, 6].

Most traditional stroke rehabilitation programs lack exercise intensity and there is no standard evaluation tool [7]. The intensity of exercise refers to work rate or metabolic needs which could be quantified as heart rate, rating of perceived exertion, rate of oxygen consumption and walking speed [8–14]. However, it would be difficult for stroke survivors to increase their work rate or metabolic needs and walking speed because of the underlying disease and the risk of fall. The intensity of exercise for stroke survivors could be quantified as the number of gait repetitions although it is a crude measure of intensity [15]. If the electromechanical assisted gait provided the uniform repetitive leg motion during the intervention, the number of gait repetitions should be in direct proportion to intervention time and we could define the intervention time as exercise intensity for electromechanical assisted gait training.

Those electromechanical-assisted gait training is known to be effective for acute and sub-acute stroke patients [6–8], and meta-analysis suggests that the patients in the first three months after a stroke and those who are not able to walk should seem to benefit the most [16]. However, several studies have reported that electromechanically assisted gait training can improve gait function in patients with chronic stroke [4–6, 16].

**Fig. 1:**
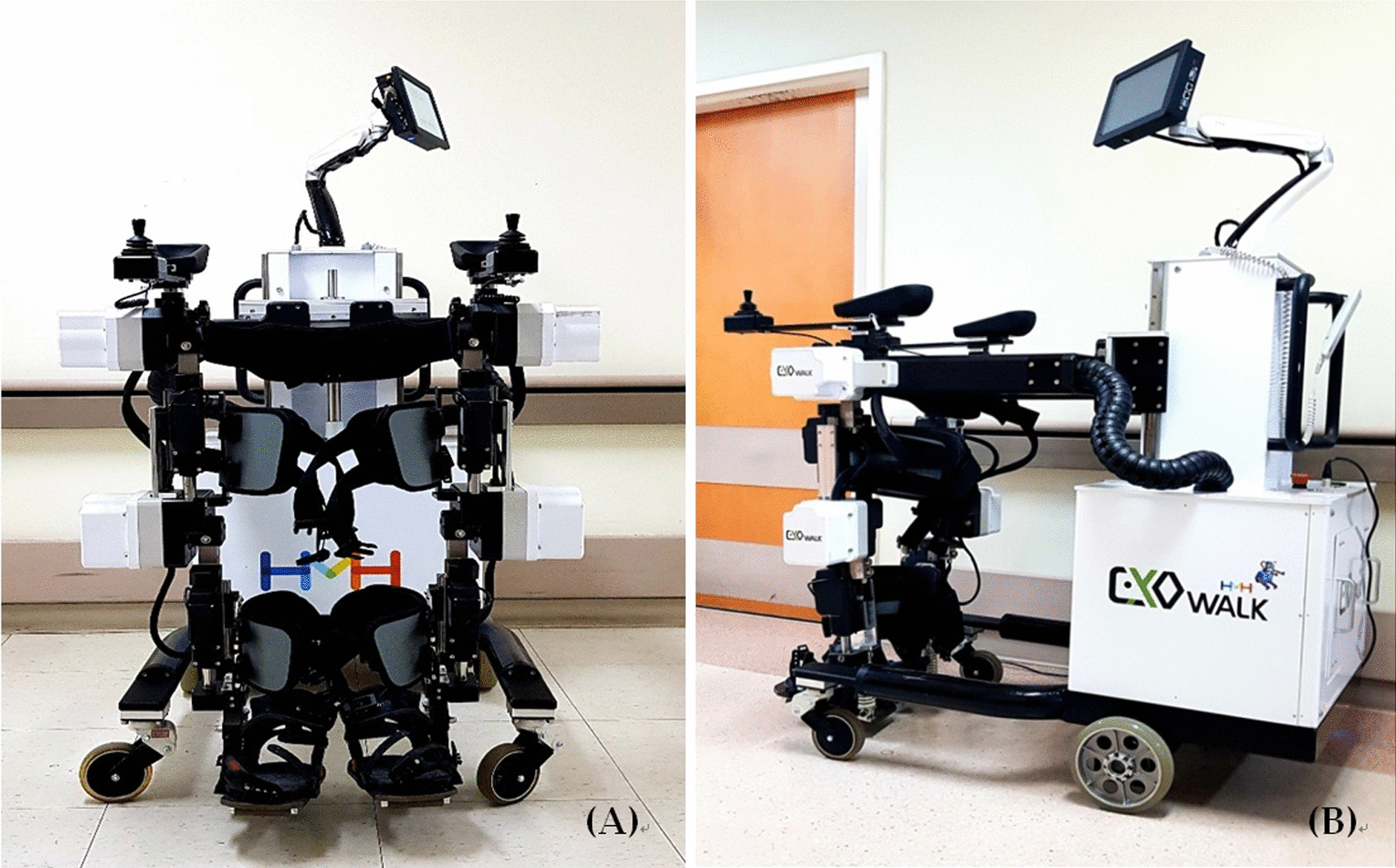
Exowalk.®, HMH Co., Ltd: **a** anterior view. **b** lateral view

Recently, electromechanical-assisted gait training by Exowalk**®** (Fig. 1) improved walking in chronic stroke patients although it was not superior to conventional therapy [17]. Exowalk**®** was developed for patients with gait difficulty to perform gait training by providing the normal gait pattern of a healthy person. It has a design as an exoskeleton and actualizes walking by the patient because most of the device’s components are located dorsally on the patient, including motorized wheels for the control of device speed and direction. This design provides a firm standing ability and obviates the need for an additional cane or walker. A questionnaire of previous studies revealed that stroke survivors should feel increased confidence in independent gait and showed high satisfaction rates [18].

There are two randomized controlled trials (RCTs) by Exowalk**®** which were conducted to investigate the effect of electromechanical-assisted gait training with the same kind of evaluation and the same type of gait training except for gait training intervention time per session [17, 18]. The intervention time per session was set as exercise intensity, and we hypothesized that high intensity of gait training could shorten the rehabilitation period for endurable stroke survivors. The purpose of this study was to assess the effect of electromechanical-assisted gait training intensity on the walking ability in patients over 3-month post-stroke.

## Methods

### Data acquisition

The data for each experimental group from two former clinical trials were selected [17, 18]. Both clinical trials were RCTs and conducted with the same kind of evaluation and the same type of gait training except for the intervention time per session.

The first clinical trial (RCT 1) was intended to assess the efficacy of electromechanical-assisted gait training on the walking ability of stroke patients based on ambulatory function, muscle strength, balance, gait speed and capacity. Forty patients with stroke who could stand alone were randomly assigned to the control and experimental groups. The experimental groups underwent gait training assisted by Exowalk**®** for 30 min per session, one session per day, 5 sessions per week, for a period of 4 weeks, and the total gait training time was 600 min [18]. The second clinical trial (RCT 2) was intended to assess the efficacy of electromechanical-assisted gait training on the walking ability of stroke patients who had a stroke over 3 months previously and could walk with or without another’s assistance. Forty patients were randomly assigned to the control and experimental groups. The experimental groups underwent gait training assisted by Exowalk**®** for 60 min per session, one session per day, 5 sessions a week, for a period of 2 weeks and the total gait training time was 600 min [17]. The study provided the additional gait training for a period of 2 weeks at the end of the intervention, but the outcome measures at the end of gait training of 2 weeks were adopted in this study in order to match the total gait training time of 600 min [17].

### Participant

To minimize the selection bias, we matched the stroke duration and initial functional status of stroke patients in RCT 1 and RCT 2. The inclusion criteria of this study were revised in terms of duration since stroke onset and initial ambulatory function by FAC. The revised inclusion criteria were as follows: (1) ischemic or hemorrhagic stroke confirmed by a brain imaging study; (2) age above 19 years; (3) hemiplegia or hemiparesis confirmed by physical examination, with the ability to walk with help (FAC 2–5); (4) patients with sufficient cognitive function to control walking speed and direction; (5) stroke with onset more than 3 months previously. The exclusion criteria were the same as in RCT 1 and RCT 2 and were as follows: (1) poor cognition (unable to obey a command or a Mini-Mental Status Exam (MMSE) of less than 10); (2) trunk ataxia, inability to stand; (3) severe spasticity (Modified Ashworth Scale (MAS) Grade 3 and 4); (4) severe leg osteoarthritis, inability to walk, and (5) inability to undergo gait training.

The number of subjects in the experimental groups in RCT 1 and RCT 2 was 18 each. Two patients in RCT 1 were excluded because they had a stroke less than 3 months previously and their FAC was level 1. Four patients in RCT 2 were excluded because their FAC was level 6 and they could walk independently without help. Thus, the number of subjects included in this study was 30 persons.

### Interventions

The experimental group of RCT 1 was allocated to the low-intensity (LI) group with the electromechanical-assisted gait training for 30 min per session. The experimental group of RCT 2 was allocated to the high-intensity (HI) group and performed the same intervention for 60 min per session. Intervention and evaluation were performed by different physiotherapists with 5 years or more of experience, in order to increase reliability by minimizing the measurement error. This was a single-blind clinical trial in that outcome assessors were blind. At enrollment, patients were instructed not to reveal their allocation arm to the outcome assessor. The researcher who performed the randomization and data analyses was not involved in assessment and training.

### Measurements and analyses

Both LI and HI groups had the same outcome measures. The primary outcome was the change of FAC [19]. Secondary outcome were the changes of Rivermead Mobility Index (RMI) [20], 10**-**m walk test (10MWT) [21], 6-min walk test (6MWT) [22], Motricity Index (MI) [23], Berg Balance Scale (BBS) [24], and Modified Barthel Index (MBI) [25]. All assessments were conducted within 1 week before and after gait training.

FAC represented the walking ability on a 6-point scale. Walking ability was assessed by the need for walking assistance [19]. RMI was a test to assess mobility based on 15 items ranging from turning over in bed to running based on a question format of Yes or No [20]. The 10MWT was used to measure the walking ability of the subjects which was used to measure walking velocity based on average speed (meter/s) after three times of 10-m walking [21]. The 6MWT was used to measure walking capacity, which was recorded as the distance calculated by the number of repetitions in a 30-m cycle for 6 min [22]. MI was calculated only for the lower legs to represent muscle strength. The total MI score was recorded in a range of 0 to 100 points, with the higher numbers representing good muscle strength [23]. BBS testing is a clinical measurement method used to measure the risk of falls in stroke patients with the higher the score, the better the balance ability [24]. MBI is the ADL outcome measures, and consisted of 10 items: feeding, personal hygiene (grooming), bathing, dressing, toilet transfer, bladder control, bowel control, chair/bed transfers, stair climbing, and ambulation [25]. All assessments were conducted within a week pre-gait training and post-gait training, and the patients used the same walking assistance for the assessment of 10MWT and 6MWT during pre and post-gait training. All values are presented as mean and standard deviation (mean ± SD).

Continuous data were compared using the t-test, binary data using a χ^2^ test, to compare the data between the LI and HI groups. The significance of changes between pre-gait training and post-gait training in each group was assessed by using a paired Wilcoxon signed-rank test. Age was different between the two groups (Table 1) and we analyzed the data between groups with covariance of age (Table 3). Mixed model ANCOVA adjusted by the effect of age between groups (LI versus HI group) and within group (pre versus post-gait training time) showed the same result (Table 4). All statistical analyses were done using SPSS Version 22.0 (SPSS, Chicago, IL, USA). Statistical significance level was set at *p* < 0.05.

**Table 1:** The baseline characteristics of the low-intensity and high-intensity groups

	LI group (*n* = 16) 30 min (4 weeks)	HI group (*n* = 14) 60 min (2 weeks)	*p* value^*a*^
Age (years)	46.94 ± 15.61	61.86 ± 11.33	0.006
*Sex*			0.282
Male, n (%)	10 (62.5%)	6 (42.9%)	
Female, n (%)	6 (37.5%)	8 (57.1%)	
Post-stroke duration, days	475.31 ± 411.42	511.07 ± 292.91	0.789
*Stroke* *type*			0.431
Ischemic, n (%)	8 (50%)	9 (64.3%)	
Hemorrhagic, n (%)	8 (50%)	5 (35.7%)	

SD, standard deviation; LI, low intensity; HI, high intensity

^a^T-test and χ² test SD

## Results

Sixteen patients were included in LI group, and 14 patients were included in HI group in this study. Age in HI group was significantly greater than in LI group (Table 1). Therefore, the data was adjusted by the effects of age using the analysis of covariance (ANCOVA) and least-square mean (LS mean) represents the average covariate value (Table 3).

**Fig. 2:**
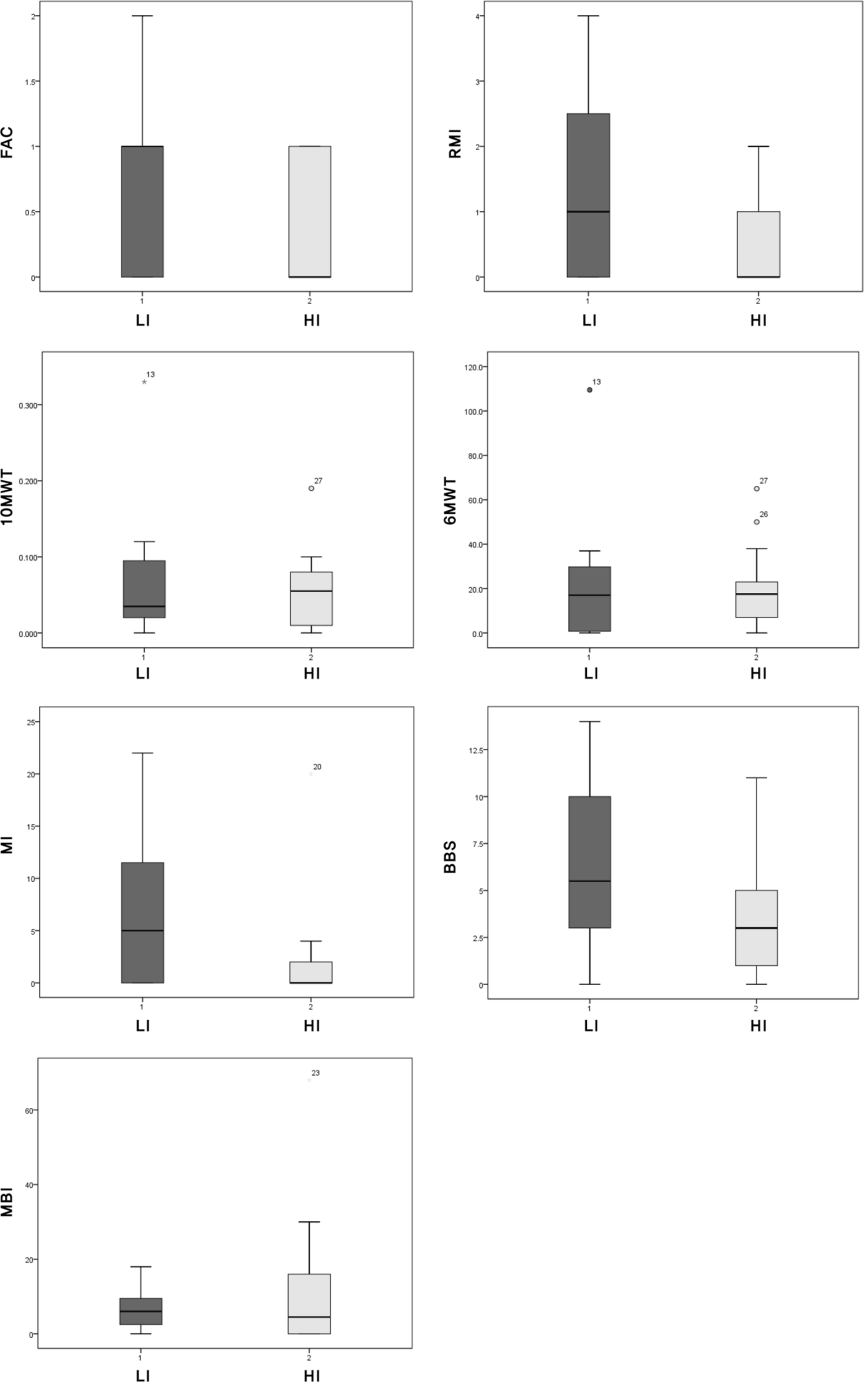
The change of outcome measures after intervention with low-intensity (LI) and high-intensity (HI) gait training

All outcome measures pre-gait training were not different statistically between LI and HI group (*p* > 0.5). In the LI group, the FAC was 3.19 ± 1.04 pre-gait training and 3.81 ± 1.22 post-gait training, and the FAC improved significantly post-gait training (*p* = 0.004). In the HI group, the FAC was 3.43 ± 1.09 pre-gait training and 3.86 ± 1.17 post-gait training, and the FAC also improved significantly post-gait training (*p* = 0.014). Most secondary outcomes in the LI and HI group improved significantly post-gait training (Table 2, Fig. 2). Whereas 10MWT, 6MWT, BBS, and MBI in HI group were improved significantly post-gait training, RMI and MI were not improved significantly (Table 2). The change of FAC after gait training was 0.63 ± 0.61in the LI group and 0.43 ± 0.51in the HI group. The changes of FAC were not different between the LI and HI groups (*p* = 0.200). The change of RMI in the LI group was greater than that in the HI group statistically (*p* < 0.015), but it was not reach the level of minimal clinically important difference (Table 3). Mixed model ANCOVA adjusted by the effect of age between groups (LI versus HI group) and within each group (pre versus post-gait training time) showed the same result (Table 4).

**Table 2:** The outcome measures before and after gait training

	LI group (*n* = 16) 30 min (4 weeks)			HI group (*n* = 14) 60 min (2 weeks)		
	Pre-gait training	Post-gait training	*P* value	Pre- gait training	Post-gait training	*p* value
FAC	3.19 ± 1.04	3.8 ± 1.22	0.004	3.43 ± 1.09	3.86 ± 1.17	0.014
RMI	5.38 ± 2.39	6.87 ± 2.60	0.003	7.71 ± 3.73	7.93 ± 3.47	0.317
10MWT	0.47 ± 0.83	0.72 ± 1.56	0.002	0.38 ± 0.28	0.43 ± 0.29	0.010
6MWT	89.87 ± 68.64	108.34 ± 74.10	0.017	105.07 ± 90.65	125.00 ± 94.68	0.006
MI	44.44 ± 14.27	51.25 ± 12.94	0.003	54.29 ± 18.97	56.36 ± 17.57	0.068
BBS	28.38 ± 13.52	34.8 ± 13.85	0.001	31.79 ± 18.81	35.21 ± 18.25	0.002
MBI	58.19 ± 16.98	64.56 ± 17.34	0.001	63.86 ± 20.59	75.21 ± 14.55	0.012

*P*-value by Wilcoxon signed-rank test between pre-gait training and post-gait training

Numbers are mean ± standard deviation

FAC, Functional Ambulation Categories; RMI, Rivermead Mobility Index; 10MWT, 10-meter walk test; 6MWT, 6-minute walk test; MI, Motricity Index; BBS, Berg Balance Scale; MBI, Modified Barthel Index

**Table 3:** The change of outcome measures before and after gait training and the difference of values between the low-intensity and high-intensity group

	LI group (*n* = 16) 30min (4 weeks)		HI group (*n* = 14) 60min (2 weeks)		*p* value
	Mean ± SD	LS mean ± SE	Mean ± SD	LS mean ± SE	
FAC	0.63 ± 0.61	0.68 ± 0.15	0.43 ± 0.51	0.36 ± 0.16	0.200
RMI	1.50 ± 1.41	1.54 ± 0.30	0.36 ± 0.63	0.30 ± 0.32	0.015
10MWT	0.24 ± 0.73	0.27 ± 0.14	0.56 ± 0.51	0.02 ± 0.15	0.284
6MWT	20.96 ± 27.31	18.28 ± 6.26	21.35 ± 18.39	24.41 ± 6.75	0.537
MI	6.81 ± 6.55	6.70 ± 1.63	2.07 ± 5.32	2.19 ± 1.76	0.090
BBS	6.50 ± 4.41	6.39 ± 1.06	3.43 ± 3.20	3.55 ± 1.14	0.099
MBI	6.38 ± 4.89	11.36 ± 18.73	8.52 ± 3.42	8.89 ± 3.69	0.946

*P*-value by the analysis of covariance of age between LI and HI groups

Numbers are mean ± standard deviation

LS mean, least square mean; SE, standard error; FAC, Functional Ambulation Categories; RMI, Rivermead Mobility Index; 10MWT, 10-meter walk test; 6MWT, 6-minute walk test; MI, Motricity Index; BBS, Berg Balance Scale; MBI, Modified Barthel Index

**Table 4:** Mixed model analysis of covariance (ANCOVA) between time (pre versus post-gait training) and group (low versus high-intensity)

	Sum of Squares	df	Mean square	F	*p*
*FAC*					
Time	0.010	1	0.010	0.059	0.811
Group	0.292	1	0.292	0.242	0.627
Time*Group	0.006	1	0.006	0.036	0.851
*RMI*					
Time	0.374	1	0.374	0.528	0.474
Group	38.731	1	38.731	4.486	0.044
Time*Group	5.100	1	5.100	7.192	0.012
*10MWT*					
Time	0.007	1	0.007	0.043	0.837
Group	0.353	1	0.353	0.427	0.519
Time*Group	0.185	1	0.185	1.221	0.279
*6MWT*					
Time	1594.689	1	1594.689	4.710	0.039
Group	6727.920		6727.920	1.037	0.318
Time*Group	185.851	1	185.851	0.549	0.465
MI					
Time	4.058	1	4.058	0.219	0.644
Group	91.715	1	91.715	0.349	0.560
Time*Group	42.347	1	42.347	2.280	0.143
*BBS*					
Time	7.429	1	7.429	0.995	0.328
Group	343.204	1	343.204	1.441	0.241
Time*Group	27.710	1	27.710	3.711	0.065
*MBI*					
Time	53.713	1	53.713	0.657	0.425
Group	828.643	1	828.643	3.216	0.084
Time*Group	0.387	1	0.387	0.005	0.946

2 × 2 Mixed model ANCOVA adjusted by the effect of age

Type III Sum of Squares; Df, Degrees of freedom; MS, Mean Square; FAC, Functional Ambulation Categories; RMI, Rivermead Mobility Index; 10MWT, 10-meter walk test; 6MWT, 6-minute walk test; MI, Motricity Index; BBS, Berg Balance Scale; MBI, Modified Barthel Index

## Discussion

This study was conducted to assess the effect of electromechanical-assisted gait training intensity on walking ability in stroke patients. Generally intensity of exercise was defined as percent of heart rate maximum and high-intensity exercise could be more potent stimulus in enhancing walking competency in stroke survivors [12–14]. However, stroke is the second most common cause of death after ischemic heart disease and a strong cause of difficulties in walking [31, 32]. During gait training for the stroke patients who had walking difficulties, it was impractical to apply high-intensity gait training of over 70–95% heart rate maximum [12–14]. In this study, the intensity of gait training was defined as the intervention time per session because electromechanical-assisted gait training could provide repetitive leg motion during the intervention and the number of repetitions could be determined according to intervention time and gait speed. The speed of electromechanical-assisted gait training was set by 10MWT pre-gait training and adjusted by the clinician during the period of the clinical trial. Because there were no differences of 10MWT between LI and HI group in both pre and post-gait training, the number of repetitive leg motions should not be different within the same intervention time between groups, either. The intervention time of gait training per session was 60 min in HI group. Thus, the number of repetitions per session in HI group was twice those in LI group. Because the improvement of walking ability in LI and HI group was not different after matching the inclusion criteria and total intervention time, we suggested that intensive gait training could shorten the gait training period and the clinician could customize the training program according to the patient training abilities.

Mehrholz et al. [16] investigated 36 trials of electromechanical-assisted gait training involving 1472 participants and concluded that electromechanical-assisted gait training in combination with physiotherapy increased the odds of participants becoming independent in walking, but did not significantly increase their walking velocity or walking capacity. However, they interpreted the results with caution, because some trials investigated people who were independent in walking at the start of the study, and they found differences between the trials in terms of the duration of intervention and frequency. It is still uncertain what is the most effective frequency and intervention time of electromechanical-assisted gait training.

Some studies applied the intervention time for around 30 min per day or session [26–28] because it is a tolerable exercise time for stroke patients. However, a few studies tried the intervention time for 60 min per session [29, 30] and it was tolerable for chronic stroke patients who could walk with or without help. Bang and Shin [29] reported that chronic stroke patients who had robot-assisted gait training for 60 min a day, 5 days a week, for 4 weeks, showed better walking abilities and balance than those who had treadmill gait training. However, Stein et al. [30] reported that robotic therapy for ambulatory stroke patients with chronic hemiparesis using a robotic knee brace resulted in only modest functional benefits that were comparable to those from a group exercise intervention, although they did the robot therapy for 60 min a day, 3 days a week, for 6 weeks. We also tried intensive 60-min gait training to get a better result by increasing the intervention time per session and found that the 60-min electromechanical-assisted gait training improved ambulatory function as much as the physical therapist-assisted gait training although the improvements did not meet the minimal clinically important difference (MCID) [17]. The change of RMI in HI group in RCT 2 was 0.17 ± 0.70 and it was not included in the result because MCID of the change of RMI was 3.0 [33]. In this study, the change of RMI was 1.54 ± 0.30 in LI group and 0.30 ± 0.32 in HI group, and both did not meet MCID although the difference between groups were significant (Table 3).

Stroke patients who could walk with another’s assistance (FAC 2, 3) or requiring help (FAC 4, 5) were included in this study. Chronic stroke patients who could walk with help participated actively in RCT 2 because they wanted walk well. And they could tolerate 60-min gait training and eagerly wanted to obviate the need for a cane or another’s assistance. This study had new inclusion criteria of the chronic patients who had stroke duration over 3 months and those who could walk help. The patients of FAC 6 in RTC 2 who could walk independently were excluded, because they could walk without help and did not expect further improvement of FAC. We intended to find out whether we could shorten the gait training period if providing the electromechanical-assisted gait training intensively, because Exowalk® could provide unlimited repetition and most accurate motion. This study found the same improvement of walking ability after 2 or 4 weeks of gait training if providing the same total intervention time. Electromechanical-assisted device in this study provide repetitive training with symmetric gait motion for stroke patients. Improvement of gait symmetry was achieved after symmetrical walking training and it related to the balance [34]. We needed to investigate the quantitative gait analysis.

We tried to compared the data of LI and HI group to control group in each LI and HI group after applying the revised inclusion criteria. In the previous RCT 1 study of LI group [18], we analyzed the data with covariance of age and stroke duration which were different between control and experimental group before gait training. After adjusting the data with covariance of age and duration, the change of all outcomes was not different between groups. In the previous RTC 2 study of HI group [17], the change of both primary and secondary outcomes were not different between control and experimental group although they improved significantly after gait training within each groups. In this study by the revised inclusion criteria, the change of all outcomes after gait training was not different, either. However, the number of patients by the revised inclusion was small and we need statistical adjustment. This study was conducted by single electromechanical assisted gait trainer of Exowalk**®** and we need to review the articles of LI and HI gait training because the meta-analysis by many electromechanical assisted gait trainers would be more informative.

## Limitations

This study was conducted by combining two separate RCTs to evaluate the effect of gait training intensity on walking ability instead of designing a prospective study because it was difficult to assign the different walking capacity patients to different intervention time randomly. Because we had two types of gait training regimen, we compared the result in advance and tried to find the effect of intensive gait training. Although two RCTs had the same protocol of intervention and evaluation, they had different inclusion criteria and were vulnerable to selection bias.

No power calculations were performed because sample data for this study from two previous trials. we set intervention time as exercise intensity because it was difficult to increase work demand by walking in the patients with walking difficulties. We need to investigate the carry over compare the effect by evaluating outcome measures at 4 weeks in the HI group, because we shortened the intervention period by intensive gait training and expected the improvement to be lasted.

## Conclusions

We could expect the same improvement of walking ability after LI or HI gait training, if we provided the same total gait training time. We could shorten the gait training period by providing the gait training intensively and we could customize the training program according to the patient training abilities.

## Data Availability

The data presented in this study are available on reasonable request from the corresponding author.

## Abbreviations

MMSE: Mini-Mental State Examination
FAC: Functional Ambulation Categories
RMI: Rivermead Mobility Index
10MWT: 10Meter walk test
6MWT: 6Minute walk test
MI: Motricity Index
BBS: Berg Balance Scale
MBI: Modified Barthel Index
